# Acute Physiological and Thermoregulatory Responses to Extended Interval Training in Endurance Runners: Influence of Athletic Performance and Age

**DOI:** 10.1515/hukin-2015-0123

**Published:** 2015-12-30

**Authors:** Felipe García-Pinillos, Víctor Manuel Soto-Hermoso, Pedro Ángel Latorre-Román

**Affiliations:** 1 Faculty of Education Sciences. Department of Didactics of Corporal Expression. University of Jaén (Spain). Jaén (Spain); 2 Faculty of Sport Sciences. Department of Physical Education. University of Granada (Spain). Granada (Spain)

**Keywords:** training prescription, long-distance runners, high-intensity intermittent training

## Abstract

This study aimed to describe the acute impact of extended interval training (EIT) on physiological and thermoregulatory levels, as well as to determine the influence of athletic performance and age effect on the aforementioned response in endurance runners. Thirty-one experienced recreational male endurance runners voluntarily participated in this study. Subjects performed EIT on an outdoor running track, which consisted of 12 runs of 400 m. The rate of perceived exertion, physiological response through the peak and recovery heart rate, blood lactate, and thermoregulatory response through tympanic temperature, were controlled. A repeated measures analysis revealed significant differences throughout EIT in examined variables. Cluster analysis grouped according to the average performance in 400 m runs led to distinguish between athletes with a higher and lower sports level. Cluster analysis was also performed according to age, obtaining an older group and a younger group. The one-way analysis of variance between groups revealed no significant differences (p≥0.05) in the response to EIT. The results provide a detailed description of physiological and thermoregulatory responses to EIT in experienced endurance runners. This allows a better understanding of the impact of a common training stimulus on the physiological level inducing greater accuracy in the training prescription. Moreover, despite the differences in athletic performance or age, the acute physiological and thermoregulatory responses in endurance runners were similar, as long as EIT was performed at similar relative intensity.

## Introduction

Taking into consideration that success in endurance running involves both aerobic and anaerobic metabolism (Brandon and Boileau, 1992), endurance runners use different training methods (Hawley et al., 1997; Rabadán et al., 2011). As mentioned by Midgley et al. (2007), endurance runners often seek the most effective training methods to enhance performance, however, the most effective method is not always the healthiest one. The incidence of running-related injuries on an annual basis is high, occurring in 40–50% of runners (Fields et al., 2010) and, although it is widely accepted that injuries in endurance runners are multifactorial, it is also well known that running-related injuries are often attributable to training errors (Nielsen et al., 2012). The knowledge about every possible effect of a particular training protocol on the athlete plays a key role in the proper training prescription, which means that a further description of the impact of most typical running exercises on endurance runners is necessary.

As for the physiological response, abundant information is available, even though most scientific papers have focused on acute response to continuous running exercise from distances as short as 400 or 1600 m (Canfield et al., 2013), to long-distance running as a half-marathon (Piacentini et al., 2014). However, to the best of our knowledge only a few studies have analysed the physiological impact of intermittent training in runners (Gorostiaga et al., 2010; Vuorimaa et al., 2000), but none of them has worked with endurance runners during field-based extended interval training (EIT).

Regarding the thermoregulatory response, in spite of being well known the influence of core temperature (Tc) on athletic performance (Drust et al., 2005), to date, there is a lack of literature reporting the evolution of Tc during an intermittent running exercise in endurance runners.

Taking the above information into account, this study focused on the acute physiological and thermoregulatory responses to a common running exercise in endurance athletes. Therefore, the main objectives of this study were: i) to describe the acute impact of extended interval training (EIT) at physiological and thermoregulatory levels, and ii) to determine whether athletic performance and age influence acute physiological responses in endurance runners.

## Material and Methods

### Participants

Thirty-one recreational male endurance runners, with a minimum experience of 6 years of training and competition (age = 28.26 ± 8.27 years, body mass index [BMI] = 22.24 ± 2.50 kg/m^2^, and maximal oxygen uptake [VO_2max_] = 58.7 ± 4.50 ml·kg^−1^·min^−1^), voluntarily participated in this study. Most participants performed 6–7 running workouts per week, lasting more than 60 min. The athletes trained regularly and had no history of injury in the 3 months before the study. More information about the participants is shown in Table 1. The study was conducted during the competitive season.

After receiving detailed information on the objectives and procedures of the study, each subject signed an informed consent form to participate, which complied with the ethical standards of the Declaration of Helsinki (2013). The subjects could withdraw from the study at any point. The study was approved by the Ethics Committee of the University of Jaen (Spain) and was conducted following the European Community’s guidelines for Good Clinical Practice (111/3976/88 of July 1990) and the Spanish legal framework for clinical research on humans (Real Decreto 561/1993 on clinical trials).

### Procedures

Subjects were tested individually on 2 occasions. First, during a preliminary session, an anthropometric assessment and an incremental running test were carried out. The following anthropometric variables were evaluated: body height (m) measured with a stadiometer (Seca 222; Hamburg, Germany), body mass (kg) recorded with a Seca 634 scale (Hamburg, Germany), and the BMI (body mass [kg]/ height [m^2^]). As for the running test, the Léger test (Léger et al., 1988) was performed, through which VO_2max_ could be estimated and which consisted of 20 m sprints with increasing speed in each run, indicating the pace with audible signals. The VO_2max_ was calculated based on the speed that the participant reached in the last sprint through the following equation (Léger et al., 1988): VO_2_ (ml·kg^−1^·min^−1^) = 5.857 × velocity (km/h) − 19.458.

The second session was performed 7 days after the preliminary session on an outdoor running track (lane 1) (temperature = 17.16 ± 5.81 ºC, relative air humidity = 62.65 ± 16.06%). Subjects were instructed to avoid strenuous exercise 72 hours before the training protocol. The runners were free to drink water during the running protocol. Before EIT, the athletes performed a warm-up, which consisted of 5–10 min of continuous running at a comfortable speed and 10 min of general exercises (high skipping, leg flexions, jumping exercises, and short bursts of acceleration). Then the participants began the EIT protocol, which consisted of 12 runs of 400 m, grouped into 4 sets of 3 runs, with a passive recovery period of 1 min between runs and 3 min between sets (4 × 3 × 400 m). Interval training is used in the physical preparation of almost all endurance athletes (Gorostiaga et al., 2010; Vuorimaa et al., 2006) and is characterized by efforts lasting from 60 to 90 s with an intensity of 85–100% of maximal aerobic speed and with a high volume.

Between each 400 m run, the rate of perceived exertion (RPE) on the Borg Scale (Borg, 1982) was recorded together with the peak heart rate achieved and the recovery heart rate at 1 min (HRpeak and HRrec, respectively), using the Garmin Forerunner monitor 405 (KS, USA). As indicated by Daanen et al. (2012), although the HRrec is generally expressed in absolute terms (bpm), it may be useful to express it relatively to the HRrec (ie, the difference between resting and maximal heart rate) to minimize interpersonal differences. Based on this, the difference between the HRpeak and HRrec at 1 min was calculated and was called heart rate reserve (HRR, in bpm). Also the tympanic temperature as an index of Tc (Brandon and Boileau, 1989; Roth et al., 1996) was recorded after each run. For this purpose, an infrared tympanic thermometer (ThermoScan® IRT 6020, Braun™, Germany) was used according to the manufacturer’s guidelines. This device had been previously used and found reliable (Kocoglu et al., 2002). The time to cover each 400 m run (s) was also recorded although the time used for subsequent analysis was the average of the whole EIT protocol (T400m). Moreover, blood lactate (BLA, mmol.l^−1^) was recorded after the last run of each set, and for this purpose, a portable lactate analyzer Lactate-Pro (Arkray, Inc.) was used.

### Statistical Analysis

Descriptive statistics are represented as mean (SD), as well as percentages (%). Tests of normal distribution and homogeneity (Kolmogorov-Smirnov and Levene’s) were conducted on all data before analysis. Analysis of repeated measures (ANOVA) was performed comparing the scores in analyzed variables throughout EIT. A Pearson correlation analysis between the increments (12^th^ run – 1^st^ run) of analyzed variables was used. Finally, k-means clustering was performed according to the T400m performance, and another according to the age of participants. Analysis of covariance (ANCOVA) was performed between the created groups, using VO_2max_ and the BMI as covariates. The level of significance was set at p ≤ 0.05. Data analysis was performed using SPSS (version 21, SPSS Inc., Chicago, Ill).

## Results

**Figure 1:**
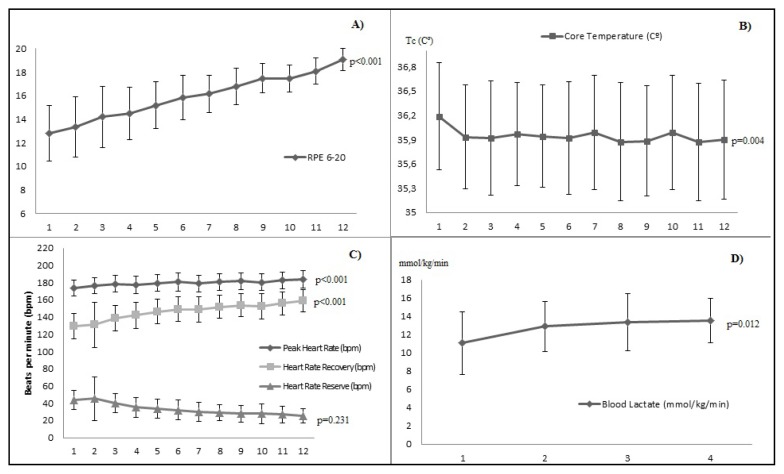
Changes of the analysed variables throughout extended interval training (4 × 3 × 400 m): A) rate of perceived exertion in a 6–20 scale; B) core temperature in Celsius degrees (ºC); C) peak heart rate, heart rate recovery and heart rate reserve, all of them in beats per minute (bpm); D) blood lactate in millilitre per kilogram per minute.

Figure 1 shows the results obtained in the variables analysed throughout EIT (4×3×400 m). Significant differences were found in the RPE (p<0.001), Tc (p=0.004), the HRpeak and HRrec (p<0.001), and BLA (p=0.012) while the HRR remained unchanged (p=0.231).

The cluster analysis performed according to the T400m (Table 2) leads to distinguish between athletes with a higher sports level (HLG, T400m=73.07 s, n=23), and those with a lower one (LLG, T400m=84.91 s, n=8). The one-way analysis of variance reveals that both HLG and LLG show significant differences (p<0.001) in VO_2max_. Nevertheless, the results obtained show that no significant differences were observed in the remaining variables considered. A cluster analysis was also performed according to the age of participants (Table 2), obtaining an older group (OG, age=38.50, n=10) and a younger group (YG, age=23.52, n= 21). The same statistical procedure was used and significant differences between the OG and YG were found only in the BMI (p= 0.006) and VO_2max_ (p= 0.030).

The Pearson correlation analysis performed for the whole group (n=31) between the increases of analysed physiological variables (Δ, results obtained in the first 400 m run – results obtained in the last 400 m run) and the rest of monitored variables (T400m, age, BMI and VO_2max_) shows a significant correlation (r=0.503, p=0.004) between ΔHR and ΔHRrec, as well as a significant and negative correlation between ΔHRrec and the ΔHRR (r=−0.832, p<0.001). Besides, age correlates significantly with the BMI (r=0.611, p<0.001) and VO_2max_ (r=−0.497, p=0.004), while T400m correlates with VO_2max_ (r=−0.799, p<0.001).

## Discussion

One of the aims of this study was to describe the impact of EIT (typical workout for endurance runners) on a physiological and thermoregulatory level. As pointed out in the introduction, training prescription’s errors play a key role in the high incidence rate of running-related injuries (Fields et al., 2010). Therefore, a further knowledge about the acute response to the most common running exercises is needed, and it will lead to a better understanding of the impact of each training stimuli on endurance runners, improving the accuracy in the training prescription. As far the authors know, no previous studies had focused on describing the evolution of physiological variables during field-based EIT in recreationally trained endurance runners. In this regard, the results obtained in the current study provide a detailed description (run by run) of commonly used variables in daily activity for athletes and coaches such as the RPE, heart rate (HRpeak, HRrec and HRR) or BLA, and one more variable not widely used but could be measured easily during any type of training with endurance runners i.e. the Tc.

The physiological variables changed in a logical way, similarly to previous studies (Gorostiaga et al., 2010; Vuorimaa et al., 2006), increasing throughout the training protocol, reaching very high intensity levels in each one of them (RPE: 18.36 ± 0.97; HRpeak: 182.20 ± 9.62 bpm; HRrec: 155.43 ± 13.07 bpm; and BLA: 13.55 ± 2.41 mmol·l^−1^). The high levels of BLA suggest that anaerobic glycolysis is extensively activated during these types of exercise, preferentially in type II muscle fibres (Green, 1978). There are numerous contrasting views of the physiological effects of lactate and its role on post exercise metabolism. On the one hand, there is a clear association between the production of lactate and muscular fatigue (Facey et al., 2013). High BLA is known to reflect a decreased muscle pH level and a concomitant fall in the force output of muscle contraction (Hultman and Spriet, 1986). On the other hand, muscle is now considered to be a consumer of lactate (Facey et al., 2013). As indicated by Brooks (2009), a relatively high BLA level (>10 mmol·l^−1^) may also indicate high lactate consumption in working skeletal muscles, which may in fact enhance muscular performance. Anyway, BLA is of interest as it is considered to indirectly reflect the degree of anaerobic glycolysis activation (McCartney et al., 1986) and, consequently, the intensity of the previous work.

As for the thermoregulatory response, Tc showed a significant reduction throughout EIT, reaching immediately after the last 400 m run an average of 35.89 ºC. The results reported are opposing to previous studies which found an increase in Tc after different endurance races such as a half-marathon (Lee et al., 2010) or 4 km cross-country racing (Hunter et al., 2006). Probably, the fact that these studies were performed in a hot environment and data were collected during continuous running can explain this difference. Additionally, a wide variety of methods are available to measure Tc what makes the comparison of results difficult. Nevertheless, among the non-invasive sites for Tc measurement, tympanic temperature probably has the strongest association with Tc (Folk et al., 1998; Lee et al., 2010). To our knowledge, no previous studies had used intermittent running exercises under moderate environment with endurance runners. Just a few studies (Drust et al., 2005; Duffield et al., 2009) performed in dynamic sports allow for a comparison owing to the intermittent nature of these sport modalities. Both studies associated the increase in Tc with impairment in athletic performance, a situation not found in the current study in which the athletic performance, in terms of time required to complete 400 m runs, remained unchanged. As indicated by Moran and Mendal (2002), an increase in Tc during exercise results in overloading the cardiovascular and metabolic system. In a previous study, Pujol et al. (1994) found a decrease in Tc after a marathon in relation to resting values. The authors suggested that the lack of an increase in Tc during prolonged running could be an indicator of running economy, highly developed ability in endurance runners, and its relationship with the thermoregulation is not totally clear (Joyner et al., 2011). As concluded by Cheuvront and Haymes (2001) in a review, numerous individual and external factors can influence Tc, however, common consensus is lacking. Environmental conditions, dehydration and a metabolic rate, as well as gender, are commonly referenced as limiting factors in thermoregulatory control (Cheuvront and Haymes, 2001; Lee et al., 2010; Lim et al., 2008). In the current study, Tc baseline values at rest (pretest) were normal (36.43 ºC) (Folk et al., 1998) and, as earlier mentioned, the running exercise was conducted under moderate environmental conditions, so more research is needed on Tc in experienced athletes during intermittent exercise under different environmental conditions.

Determining whether athletic performance and age effect acute physiological and thermoregulatory responses in endurance runners was the other main purpose of this study. That is why the researchers decided to incorporate cluster analysis as members of the same cluster are likely to have similar responses. Regarding the first variable, athletic performance, two clusters of endurance athletes were obtained from the different magnitude of the T400 m performance (HGL and LLG). The major finding in this line was the fact that no significant differences between groups concerning physiological or thermoregulatory responses occurred. In this study, the HLG obtained VO_2max_ significantly higher than the LLG (+5.02 ml·kg-1·min-1), as well as a greater T400 m (11.84 s). Nevertheless, changes of the analysed variables throughout EIT do not show significant differences between the HLG and LLG. This means that the impact of EIT on the physiological level is the same, in spite of the differences in athletic performance, as long as the workout was performed at the same relative intensity and absolute load. No previous papers had been directly focused on this topic, however, some findings reinforce our results. Canfield and Gabel (2013) found that different distances runners (sprinters vs. endurance runners) did not differ in the HR or RPE after running two distances. In the same line, some previous studies had not observed a relationship between metabolic and physiological markers and performance during intermittent-sprint exercise (Duffield et al., 2009), or a football match (Krustrup et al., 2006).

With regard to the age effect, the major finding was the lack of differences in the physiological and thermoregulatory responses. At the same absolute load, both the OG and the YG showed similar responses, in spite of the difference in age (+14.98 years for the OG), VO_2max_ (-2.97 ml·kg^−1^·min^−1^), and the BMI (+2.36). Previous papers concluded that age could affect the HRpeak (Gellish et al., 2007), the HRrec (Daanen et al., 2012), while peak lactate levels remained unchanged (Nybo et al., 2014). The correlations obtained for the whole group between age, the BMI and VO_2max_, as well as the inter-groups (OG-YG) differences in the HRpeak (OG: 176.71, YG: 187.01), support the above rationale. Nevertheless, the lack of differences between the OG-YG according to the behaviour of analysed variables throughout EIT allows to highlight that age is not a factor that influences the acute physiological and thermoregulatory response during EIT in experienced endurance runners.

Despite its exploratory nature, this study offers some insight into acute physiological and thermoregulatory responses to a typical workout for endurance runners and the influence of variables such as athletic performance and age. These findings are limited by the field-based nature of the present study which makes impossible to control some interesting factors as hydration status, and to standardise environmental conditions.

## Conclusions

The obtained results provide a detailed description (run by run) of the physiological (HRpeak, HRrec, HRR, BLA, RPE) and thermoregulatory (Tc) responses to EIT in experienced endurance runners. The provided data help in understanding the impact of a common training stimulus on the physiological level leading to greater accuracy in the training prescription process. Moreover, despite the differences in athletic performance or age, the acute physiological and thermoregulatory responses in endurance runners are similar, as long as EIT is performed at similar relative intensity. The results suggest that the evaluation of physiological and thermoregulatory responses at the same time as running performance should be considered for monitoring of endurance athletes.

From a practical point of view, it seems quite difficult for recreational runners or coaches to afford including “gold standard” for physiological and thermoregulatory responses assessment in their daily activity. However, many athletic clubs can afford having devices as used in the current study. Hence, this study not only provides further knowledge about the impact of a typical training stimulus on endurance athletes, which plays a key role in improving performance and preventing injuries, but also includes easy-to-use tools for monitoring and controlling training adaptations in endurance runners.

## Figures and Tables

**Table 1:** Body composition (mean, SD), physical fitness (mean, SD) and daily training information (n, %) of participants.

Age (y) M (SD)		Body heigth (m) M (SD)	Body mass (kg) M (SD)	BMI (kg/m^2^) M (SD)		VO_2max_ (ml·kg^−1^min^−1^) M (SD)	
28.26 (8.27)		1.75 (0.04)	68.16 (6.70)	22.24 (2.50)		58.7 (4.50)	

Daily training							
Number of sessions per week n (%)		Duration of training sessions (min) n (%)		Perceived performance state n (%)		Training experience (y) n (%)	

5	6 (19.4%)	30–40 min	1 (3.3%)	60–79 (%)	3 (9.7%)	6–8 y	3 (10%)
6	12 (38.7%)	40–60 min	11 (36.7%)	80–100 (%)	28(90.3%)	8–12 y	9 (30%)
7	12 (38.7%)	+ 60 min	19 (60%)			+ 12 y	19 (60%)
8	1 (3.3%)						

BMI: body mass index

**Table 2:** Comparative analysis of physiological and thermoregulatory responses (mean, SD), in terms of increases and peaks reached, between the HLG and LLG, groups created according to the average performance in 400 m runs, and the OG and YG, groups created according to the age, as well as possible influence factors (BMI, VO2_max_, age, T400m, and training experience).

Variables	HLG (n=23)	LLG (n=8)	*p value*	OG (n=10)	YG (n=21)	*p*
ΔRPE	6.27 (2.53)	6.25 (2.49)	0.992	6.80 (2.57)	6.00 (2.45)	0.410
ΔTc (ºC)	−0.33 (0.68)	−0.18 (0.53)	0.574	−0.12 (0.68)	−0.37 (0.61)	0.324
ΔHRpeak (bpm)	9 (5.78)	14 (5.01)	0.038	8.50 (4.97)	11.14 (6.28)	0.254
ΔHRrec (bpm)	28.74 (10.91)	29.13 (10.76)	0.932	26.60 (12.34)	29.90 (9.97)	0.431
ΔHRR (bpm)	−19.74 (1.92)	−15.13 (3.25)	0.231	−18.10 (11.70)	−18.76 (8.12)	0.856
ΔBLa (mmol·l^−1^)	2.13 (4.68)	3.50 (2.44)	0.439	2.90 (1.94)	2.29 (4.99)	0.712
HRpeak (bpm)	183.30 (8.73)	184.75 (13.71)	0.725	176.71 (10.64)	187.01 (7.50)	0.004
Peak BLa (mmol·l^−1^)	13.96 (2.28)	12.35 (2.52)	0.103	13.22 (2.19)	13.71 (2.55)	0.606
BMI	21.64 (1.82)	23.13 (3.37)	0.136	23.63 (2.42)	21.27 (1.94)	0.006
VO2_max_ (ml·kg^−1^·min^−1^)	58.05 (2.09)	53.03 (3.20)	<0.001	54.95 (3.70)	57.62 (2.69)	0.030
Age (y)	27.30 (7.73)	31.30 (10.20)	0.247	38.50 (4.71)	23.52 (4.60)	<0.001
T400m (s)	73.07 (3.62)	84.91 (5.91)	<0.001	77.40 (6.68)	75.51 (6.84)	0.473
Training experience (y)	7.17 (1.94)	6.63 (1.59)	0.480	7.80 (0.570)	6.67 (0.393)	0.113

HLG: higher level group; LLG: lower level group; OG: older group; YG: younger group; Δ: increase according to post-pre comparison; RPE: rate of perceived exertion; Tc: core temperature; HRpeak: peak heart rate; HRrec: heart rate recovery at 1 minute; HRR: difference between HRpeak and HRrec; BMI: body mass index; VO_2max_: maximal oxygen uptake; T400m: average time in 400 m runs.
